# 2D bSSFP real-time cardiac CINE-MRI: compressed sensing featuring weighted redundant Haar Wavelet regularization in space and time

**DOI:** 10.1186/1532-429X-15-S1-P49

**Published:** 2013-01-30

**Authors:** Jun Liu, Alban Lefebvre, Michael O Zenge, Michaela Schmidt, Edgar Mueller, Mariappan S Nadar

**Affiliations:** 1 Siemens Corporate Research, Princeton, NJ, USA; 2 MR PI, Siemens AG, Erlangen, Germany

## Background

Compressed sensing promises to achieve a significant acceleration of the data acquisition in the case of real-time CINE-MRI. The major challenge is to achieve both high spatial and temporal resolution. In the current work, an alternative solution to k-t SPARSE SENSE is proposed which exploits spatio-temporal correlations beneficially.

## Methods

Sparse, incoherent sampling was implemented in a conventionally triggered 2D bSSFP real-time cardiac CINE-MRI sequence. The sampling pattern features an increasing acceleration factor towards the k-space periphery while all phase-encoding steps outside a central region were skipped on one side. Pairing of successive readouts was used to avoid eddy currents. In addition, a pseudo-random offset was applied for each time frame.

Image reconstruction was performed representing the time series of images as a 3-dimensional tensor x=[x1, x2,…, xt] and minimizing ∑_i ∑_j 1/2 \|\|D_i F(s^j⊙x_i)-y_i^j \|\|_2^2 +λ\|\|d⊙(Wx)\|\|_1.

Here, Di is the undersampling operator, F is the 2-dimensional Fourier transformation, yij are the k-space data acquired by the j-th coil element. ⊙ is the component-wise multiplication operator. Wx represent the coefficients after redundant Haar Wavelet transformation. These are compressible due to spatial and temporal correlations. d is a weighting operator that imposes strong weights to the temporal correlations. sj is the coil profile of the j-th coil which was estimated following the Eigen-vector approach applied to the temporal average of all input data. The optimization problem was solved using the Nesterov's approach together with an extension of the Dykstra's algorithm for solving the associated proximal operator.

Data acquisition was performed on a 3.0T MR scanner (MAGNETOM Skyra, Siemens AG, Erlangen, Germany) in 5 volunteers: FOV 380×255 mm^2^, matrix 224×168, TR/TE 2.9/1.26 ms, bandwidth 1395 Hz/px, flip angle 68, temporal resolution 33 ms, 38 temporal phases, 30 coils, and 10 frequency encodings were acquired for each temporal phase.

## Results

**Figure 1:**
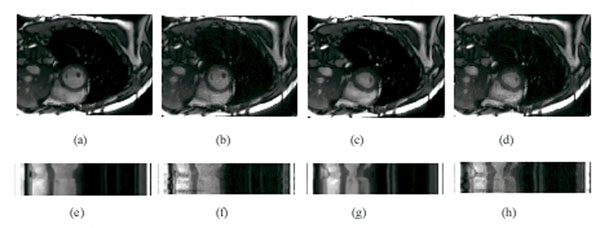
Reconstruction results by the proposed approach and k-t SPARSE SENSE. Proposed approach in (a) and (c) for phase 1 and phase 19, respectively; k-t SPARSE SENSE in (b) and (d) for phase 1 and phase 19, respectively; time plot of the proposed approach in (e) and (g); and k-t SPARSE SENSE in (f) and (h).

Figure 1 shows the results of the proposed approach in comparison to the k-t SPARSE SENSE approach. Although image reconstruction was successful in both cases, residual artifacts were observed in case of k-t SPARSE SENSE in particular along the temporal dimension. These artifacts were significantly reduced in the case of the novel solution.

## Conclusions

The current work demonstrates a significant improvement in image quality if spatio-temporal correlations are taken into account in case of real-time cardiac CINE-MRI. Since the initial experience in volunteers is promising, a comprehensive clinical evaluation is pending.

## Funding

Siemens AG

